# Physician and Parental Decision—Making Prior to Acute Medical Paediatric Admission

**DOI:** 10.3390/healthcare6030117

**Published:** 2018-09-17

**Authors:** Rebecca Barwise-Munro, Heather Morgan, Steve Turner

**Affiliations:** 1 Child Health, University of Aberdeen, Aberdeen AB24 3FX, UK; r.barwisemunro@btinternet.com (R.B.-M.); h.morgan@abdn.ac.uk (H.M.); 2 Health Services Research Unit, University of Aberdeen, Aberdeen AB24 3FX, UK; 3 Child Health, Royal Aberdeen Children’s Hospital, Aberdeen AB25 2ZG, UK

**Keywords:** admission, anxiety, child, parent, training

## Abstract

Background: The number of acute medical paediatric emergency admissions is rising. We undertook qualitative interviews with parents and clinicians to better understand what factors, other than the health status of the child, may influence decision making leading to emergency admission. Methods: Semi-structured interviews were conducted with parents; clinicians working in general practice, out-of-hours or the emergency department (referring clinicians); and doctors working in acute medical paediatrics (receiving clinicians). Results: Ten parents, 7 referring clinicians and 10 receiving clinicians were interviewed. Parents described “erring on the side of caution” when seeking medical opinion and one mentioned anxiety. Among themes seen among referring clinicians, “erring on the side of caution” was also identified as was managing “parental anxiety” and acting on “gut instinct”. Among receiving clinicians, themes included managing parental anxiety and increasing parental expectations of the health service. Conclusions: The study of parent and referring clinician decision-making prior to a hospital admission can identify “teachable moments” where interventions might be delivered to slow or even arrest the rise in short-stay acute medical admissions in Britain and other countries. Interventions could assure parents or referring clinicians that hospital referral is not required and help clinicians understand what they perceive as “parental anxiety”.

## 1. Introduction

The number of paediatric emergency hospital admissions has risen in recent years in countries including the UK [1,2], Sweden [3] and Denmark [4]. In the USA, where the healthcare system is different to most European countries, the number of children admitted to hospital (emergency and non–emergency) fell by 18% between 2005 and 2014 [5]. Where rises in admission prevalence are present, these are mostly explained by children who are admitted and discharged on the same day [1,2,6], and there is no evidence that the number of children presenting with severe illnesses is rising [1,2]. There are several possible explanations for the rise in acute medical paediatric admissions, which broadly include changes in patient/parent health-seeking behaviour and changing practice in healthcare, both in the community and in the hospital.

Parent health-seeking behaviour may have shifted towards seeking advice from healthcare professionals instead of “watchful waiting” at home for a number of reasons. Public health initiatives in the UK, for example “Helping parents spot the signs of sepsis” [7], actively encourage parents to look for signs which could suggest serious illness when their child is unwell and to seek medical attention. Although uncommon, there are high-profile stories in the media of fatal cases where parents were initially reassured that their child had a non-specific self-limiting illness before symptoms worsen with tragic consequences, and such high-profile stories may shift the confidence of some parents to accept a doctor’s reassurance that their child has a minor self-limiting illness and thus insist on referral to hospital.

Changes in healthcare practices in the UK also incorporate a number of factors which might have shifted the likelihood of a child being referred to paediatric hospital services by staff working in the community or in the hospital’s emergency department. Many hospitals now have a short stay assessment unit where children with apparently minor illnesses can be observed before either being discharged home or having further observation and in-hospital care, and the presence of these “observation units” may reduce the threshold of referring clinicians for referral to hospital [8]. Two policy changes in 2004 may have added to the number of short stay admissions: First, family doctors (also known as general practitioners GPs) opted out of on-call duties and this meant that the family doctor no longer assessed children outside of working hours and this interrupted continuity of care in the community [9]; second, in 2004, emergency departments were given a target of assessing a patient within 4 hours and this pressure to reach a decision may have reduced thresholds for referring to hospital paediatric services. In addition to these changes in infrastructure and responsibilities, there is recognition that some GPs may lack sufficient experience in child health for them to feel confident in identifying a child who can be diagnosed and managed outside of the hospital [10,11]. Finally, in today’s NHS there are non-medical staff working in the role previously filled by doctors, and these healthcare professionals, including physician assistants and advance nurse practitioners, often work to standard operating procedures which may not be well-suited to the subjective nature of assessing a child with an acute illness and, therefore, favour an approach of “if in doubt refer”.

At the same time as there have been shifts in parental health-seeking behaviour and the capacity of non-paediatric services to manage children, there have also been changes in hospital practice. In particular, admission duration has reduced by almost 50% [2] as hospitals adopt a “revolving door” policy where children are discharged home for ongoing observation by parents but with a safety net of representing to hospital. A second change has been the development of specialist services for chronic conditions and this has been associated with a reduction in admissions for asthma and diabetes [2].

When a parent or family doctor assesses a child who is not well there are at least three potential conclusions: First, the child is clearly well enough to be observed at home; second, the child is clearly sufficiently ill to require (further) medical assessment; and third, the child is not well and could be observed either at home or seen by a(nother) clinician. Better understanding of what influences decision making in the latter of these three scenarios may help develop interventions which could reduce admissions of children with self-limiting conditions.

The UK National Audit Office has estimated that 20% of all admissions could be cared for in the community [12] and in Italy there is concern that many acute paediatric admissions may be avoidable [13,14]. The rising number of admissions gives some urgency to the need to better understand how to develop service policy, design and delivery towards care in the community, despite differences in healthcare systems. A mixed methods approach is recognised as an ideal approach to understanding a complex socio-medical process, such as medical paediatric admissions [15]. The aim of this paper is to extend the quantitative results we have recently published [2] and here we report the results of our qualitative study, which explored reasons why children might be more likely to be admitted to hospital rather than be observed at home by their caregiver or primary care team.

## 2. Material and Methods

### 2.1. Study Design

This was a qualitative study where audio-recorded, semi-structured interviews were conducted with the parents of children who had had an emergency admission hospital, clinicians who refer acute medical paediatric admissions (referring clinicians) and clinicians working in an acute medical paediatric receiving unit (receiving clinicians). An emergency admission occurs when a child has an unscheduled admission to a hospital ward. Children are admitted to a hospital ward in the UK after first presenting to the emergency department or GP. In the UK, there are no hospital “walk in” clinics where acutely unwell children are assessed by paediatricians. Referring clinicians are either: GPs, who are family doctors who do not work in hospital and whose premises are generally open from 8 a.m. to 6 p.m., from Monday to Friday; Out-Of-Hours clinicians, who are GPs or nurse specialists and who are open when GP premises are closed; and emergency department staff, who are doctors and nurses in the emergency department at the hospital. Children seen and discharged from emergency departments were not included. All subjects gave their informed consent for inclusion before they participated in the study. The study was conducted in accordance with the Declaration of Helsinki, and the protocol was approved by the North of Scotland Medical Research Ethics committee (project reference 14/NS/1071).

### 2.2. Enrolment

A convenience sample of up to 10 parents, 10 referring clinicians and 10 receiving clinicians was sought; recruitment ceased if saturation was reached before 10 from each category were recruited. Parents (or caregivers) who accompanied a child who had been admitted to the paediatric assessment unit at Royal Aberdeen Children’s Hospital (RACH) with an acute medical condition within the last 24 h were invited to participate. The paediatric assessment unit is where emergency admissions are observed, and any treatment started, and where the child is discharged home within 24 h or transferred to another medical paediatric ward. There was no selection for cases which might have been managed at home instead of being admitted to hospital.

Referring clinicians were recruited from local GPs, doctors working to be consultants (i.e., specialty trainee 3 or above) in emergency department (ED) at RACH and clinicians working in the local out-of-hours service (OOH), see Supplementary Table S1 for details of these clinicians. Doctors working shifts in acute medical paediatrics at RACH, including foundation year (FY, doctors who are within 2 years of qualification), specialty trainee (ST, more senior doctors who are within 1–8 years of being a consultant) or an associated specialist (experienced doctors who are not trainees or consultants) were invited to participate to provide their perspective on why children might be admitted to hospital; see Supplementary Table S2 for more details of these receiving doctors.

### 2.3. Interviews

Interviews were designed to be short to accommodate clinical activities, took place in clinical settings and typically lasted about 15 min. Topic guides of questions for the interviews were created for the three separate groups of interviewees parents (Appendix A), referring clinicians (Appendix B) and receiving clinicians (Appendix C). The questions to parents and referring clinicians were structured to elicit background information and also what factors affected their decision making during the admission process. Questions to receiving clinicians were related to the appropriateness of the most recent admission they had seen and their overall views on what factors might influence decision making “upstream” of an acute referral. All interviews were conducted by the same individual (RBM), during February and March 2015. We attempted to include the same parent and referring clinician for a single admission, but this was not feasible for a number of reasons, including the referring clinician being based many miles from the hospital and being unavailable due to working shifts. Each interview audio-recording was transcribed and transcripts were systematically analysed. A thematic approach was taken and two researchers coded and compared emerging themes independently (RBM and HM) before discussing and synthesising the findings.

## 3. Results

### 3.1. Study Participants

Ten parents (including one father), 7 referring doctors (3 from EM, 2 from GP and 2 from OOH) and 10 receiving doctors (including 3 FY trainees, 6 ST trainees and 1 associate specialist) took part. Children of 7 of the 10 parents/caregiver interviewed had been admitted to hospital previously, of whom 1 had a cardiac condition (Tetralogy of Fallot), 1 had a condition which affected many parts of their body (including bones, bowel and kidney, “VACTERL” syndrome) and 1 had asthma. Children had been unwell between 12 h and 3 days prior to seeing the referring doctor and were aged between 5 weeks and 10 years. Seven children had been admitted to hospital previously. Parents were aged between 21 and 40 years. Four parents had expected their child to be admitted to hospital when they went to see their doctor, three were not expecting to be admitted and three were not sure whether they would be referred or not. Supplementary Table S3 presents all details of the children whose parents took part in the study.

### 3.2. Responses of Parents

The key theme to emerge was that parents would prefer to be “better safe than sorry” and err on the side of caution if they were unsure (Table 1). Four parents explained that they knew their child and were confident that their child was unwell (Table 1). Only one caregiver mentioned worrying (Parent A, Table 1).

### 3.3. Responses of Referring Clinicians

Four of the seven referring clinicians had no postgraduate training in acute medical paediatrics. Three main themes emerged from the question around generic triggers that may influence their decision to admit a child: parental anxiety, erring on the side of caution and acting on a “gut instinct” about whether a child is unwell.

#### 3.3.1. Parental Anxiety

Parental anxiety was mentioned as a factor that may influence the decision to admit by all but one of the referring clinicians.

“I think parental anxiety is always an issue, and there are some families, and that can be of any socioeconomic class, where their perception of illness is different from someone else’s perception of illness.”

(Doctor B from GP)

“…sometimes, no matter how skilled you are at reassuring people, you sometimes will find that you might be erring on the side of caution if you get that niggle on the back of your mind that the family are unhappy…”

(Dr. B from GP)

#### 3.3.2. “Erring on the Side of Caution”

Referring clinicians commonly reported that ‘erring on the side of caution’ was a driver of admission:

“…the safe thing to do there was to admit the child for a short period of observation…”

(Doctor F from ED)

“If I was uncertain, then that means I am not happy to send them home, by definition” and “…I think uncertainty is not a good position to leave yourself in, you either need to be happy to send someone home or you admit them, being somewhere in the middle is not a good place…” (Doctor A from ED) and “…I would always tend to err on the side of caution…”

(Doctor C from GP)

#### 3.3.3. “Gut Instinct”

‘Gut instinct’ was referred to by the majority of referring clinicians:

“…it’s a gut instinct thing I think, if I look at a child and I’m just not happy then I go with that…”

(Doctor C from GP)

“I think gut instinct is important, just know in yourself whether you think this child looks well or isn’t well…”

(Doctor D from OOH)

#### 3.3.4. Other Themes

Two of the three referring clinicians from the Emergency Department (ED) mentioned the waiting time target in the ED as something that may influence their decision to admit a child (Table 2). Other themes included the family’s socioeconomic status, a history of previous admissions, and the time of day (Table 2). One GP and one OOH doctor expressed uncertainty regarding their confidence in spotting a sick child. These two clinicians suggested that having more paediatric experience may increase their confidence. The age of the child was not mentioned.

### 3.4. Responses of Receiving Clinicians

There were three main themes that emerged. First, parental anxiety was recognised as a factor that may contribute to admission by all 10 of the referring clinicians (Table 3). Second, a perception that some referring clinicians may lack confidence in managing acute medical conditions and missing a potentially serious illness was expressed by all but one of the receiving clinicians (Table 3). Third, parents being more demanding of healthcare services was mentioned by half of the receiving clinicians (Table 3). Two of the receiving clinicians suggested that emergency admission might be prevented in some cases by educating parents about symptoms and conditions which may be serious and require hospital admission, and which conditions are less serious and can be managed at home or in the community. Other themes that emerged included: Distance the child lives from the hospital; concerns with social circumstances; and arriving to hospital in the evening and the season (Table 3). The views of receiving clinicians on the merits of short stay admissions was mixed (Table 3).

## 4. Discussion

This qualitative study identified factors which parents and both referring and receiving clinicians consider during the decision-making process leading to a hospital admission. The main finding was that parents often describe “erring on the side of caution”, but not anxiety, whilst referring and receiving clinicians perceived parental anxiety as being a common theme leading to referral to hospital. Additional findings were that referring clinicians also described “erring on the side of caution” and using “gut instinct” in their decision making and receiving clinicians describe increasing parental expectations of health care services in the context of their child’s illness. Work is now required to determine whether referrals might be safely avoided by helping clinicians interpret “parental anxiety” and interventions in cases where parent or referring clinician are “erring on the side of caution”.

These findings complement our quantitative findings [2], where we observe rising admission but no evidence of more severe illnesses, and suggest that the rising admissions may be due to human factors such as “erring on the side of caution”, “parental anxiety” and “gut feeling”. The number of children admitted with acute medical conditions continues to rise [1,2], but a systematic review has concluded that there is no intervention proven to reduce paediatric admissions [16]. Therefore, novel interventions framed around the themes of “human factors” should be designed to slow down or possibly arrest the rising number of children being admitted to hospital.

There are several factors that contribute to parents/caregivers and referring clinicians “erring on the side of caution”. A parent’s/caregiver’s threshold for seeking medical advice for their child may be altered by public health interventions for conditions, such as sepsis awareness programmes [7], and also by past experience of having a sick child or by being aware of a child in the community who has a serious health condition. Several factors are known to affect a primary care clinician’s decision making when referring to hospital [17] and the threshold for clinicians to err on the side of caution and refer a child to hospital is likely to be affected by recent experiences. Reflection on past clinical experiences and actions is core to medical training [18] and experiential learning has been described in a number of paediatric settings [19,20], but not acute referrals. The availability of a local short stay observation unit in particular may tip the decisional balance towards admission, and indeed a rise in short stay admissions might be seen as a successful outcome of these units. Interventions in partnership between primary and secondary care may help shift the decisional balance away from admission in some cases. Examples of possible interventions before a decision to admit is made include: Education targeted at clinical presentations increasingly found among short stay admissions (e.g., acute lower respiratory tract infection); standardised observation charts for use in community and hospital; and expert assessment of the child via video link.

Healthcare services face a challenge in supporting parents who are worried about their unwell child whilst also providing a service to the rest of the population; providing healthcare services to every unwell child is challenging. A key part of assessing children who are non-specifically unwell is close observation without intervention (“watchful waiting”) and resources such as “When should I worry” [21] are known to change parent health seeking behavior in the context of respiratory infections.

Parental anxiety was considered by clinicians as a factor which may be involved in the decision to admit a child, but was only mentioned by one caregiver. One explanation for this contrast might be a misunderstanding where parents are simply seeking reassurance that their child has a mild self-limiting illness, but the clinician cannot understand why the parent has attended and assumes that the parent is unduly worried and anxious. A study in the Netherlands describes how parents of a child with a fever expect a history to be taken and for their child to be examined but the majority were aware that their child might not need antibiotics [22]. Clarification of parental expectations (e.g., are you seeking reassurance?) at the start of a consultation would avoid any understanding.

A parent/caregiver of an unwell child might be expected to be anxious, and a parent might be considered “anxious” when they perceive their child to be considerably more unwell than the clinician’s assessment. Many children are cared for by grandparents or nursery staff whilst parents are at work, these carers may not know the child as well as the parent and may have a lower threshold for seeking medical attention and potentially being perceived as “anxious”. Parental perception that their child’s illness is different to their previous experiences of that child is known to alter a clinician’s assessment of a child’s wellbeing [23]. In our study, some parents did not expect to be referred to hospital by their doctor, which might suggest that “parental anxiety” is not present in a substantial minority of consultations and/or that, in these encounters, either the parent has not recognised their child as being particularly unwell or the clinician has assessed the child as being more unwell than they might have been. Clinical encounters where there is discordance between parent and clinician assessment are not uncommon and, in some cases, a second opinion is reasonable and could (for example) lead to an assessment the following morning in a “rapid review clinic”, rather than immediate hospital admission.

Some referring and many receiving doctors observed that lack of experience in acute medical paediatric conditions influenced the decision to refer a child to hospital, and the majority of referring clinicians had no postgraduate general paediatric training, which is not unusual for the UK [10]. Inevitably, receiving doctor’s opinions are likely to be biased by encounters with colleagues who refer cases, which are subsequently considered as potentially avoidable and not by encounters with experienced doctors in the community only rarely refer children. The Royal College of General Practitioners wants its members to have more exposure to acute paediatrics during their training, but also points out that the average preschool child has six encounters with primary care per annum [11], meaning that there is already considerable paediatric exposure in primary care. The challenge colleagues in primary care face is identifying the one case of serious illness, which occurs in an average of 200 primary care paediatric consultations [24].

Referring clinicians mentioned acting on a “gut feeling”, and the feeling that something is wrong despite a reassuring examination is highly specific for serious acute paediatric infection, but in a study from the Netherlands has low sensitivity [23]. The key difference between a gut feeling and erring on the side of caution is that in the former there is an aspect of the history, which is out of keeping with the “reassuring” examination (e.g., convulsion, lethargy, weight loss [23]), whilst erring on the side of caution implies that the clinician is simply not confident in their overall assessment. Thus the absence or presence of a gut feeling can give a respective sense of reassurance or one of alarm [25], whereas erring on the side of caution places the child, parent and clinician somewhere between reassurance and alarm.

There are some limitations to our work. First, qualitative research is not intended to be generalisable, but saturation was reached in our sample within many areas common to different groups, e.g., parental anxiety and erring on side of caution, and these findings are likely to be observed in other centres/sites/locations. Second, we were not able to interview both the parent and referring clinician involved in the same case and future research could seek to do this and thus compare and contrast parent and clinician perspectives of the same case. Third, parents and referring clinicians were not interviewed at the time of referral and there may be some recall bias in their interviews, but the saturation reached for many themes across participant types argues against this. Finally, many of the children whose parents participated had previously been admitted to hospital and their opinions may not be generalisable, and our research approach needs to be replicated elsewhere to better understand contextual factors that may have applied and to identify more generalisable phenomena.

In summary, we have identified a number of factors that may influence decision-making leading to a child being admitted to hospital, including a perception of parents being anxious and having increasing expectations of what healthcare services can offer and referring clinicians acting on gut feeling and erring on the side of caution. Our insights may be relevant to comparable European paediatric healthcare contexts. Without action, acute paediatric medical admissions will remain high or continue to rise in contemporary care models and our work provides insights into where interventions should be targeted in order to better manage children, i.e., in the community.

## Figures and Tables

**Table 1:** Quotes from parents of children admitted with an acute medical condition relating to some of the factors which may influence their decision to seek medical help.

Factors	Quotes
*Better safe than sorry*	“…I think it’s better you go and it’s just a false alarm than sitting at home and worrying…better to be safe than sorry.” (Parent A)
	“I would probably always err on caution and go to the GP if there was something I was concerned about definitely.” (Parent G)
*Knowing their children*	“Possibly if it wasn’t X, I would have maybe contacted someone a bit sooner, because my other kids, they are more visible when they are really unwell.” (Parent E)
	“…she’s never ill, so would probably be a bit different with her…” (Parent F)

**Table 2:** Quotes from referring clinicians relating to some of the factors which may influence their decision to refer a child to hospital with an acute medical condition.

Factors	Quotes
*Waiting time target*	“…if it was something that wasn’t going to be settling within the four hours that I had to play with here, I would certainly be speaking to my medical colleagues…” (Doctor F from ED)
*Socioeconomic background*	“…if there were markers of an adverse social background that would have pushed me more towards admission…” (Doctor E from OOH)
*Previous admissions*	“…if you have got a child who has had previous issues, particularly of a similar kind, that would definitely influence my admission…” (Doctor D from OOH)
*Time of day*	“…if I see them early on in the morning I am probably less inclined to admit, than if I see them at half four or something like that in the afternoon.” (Doctor C from GP)

**Table 3:** Quotes from receiving clinicians relating to some of the factors which they believe may influence parents and referring clinician’s decision to refer a child to hospital with an acute medical condition.

Factors	Quotes
*Parent expectations*	“…there is a lot of information about illnesses that are potentially lethal and parents tend to panic with the very small things…” (Doctor E)
	“I think parents nowadays are probably a wee bit more demanding than they used to be, so I don’t know whether they trust their GPs maybe as much as people did in the past and so want a second opinion quite a lot of the time.” (Doctor B)
	“I think most of the time it’s either a parent not coping with their child being unwell even if they are well enough to be at home or it’s over caution on our part.” (Doctor C)
*Experience of referring clinicians*	“The fact that quite often GPs get to become GPs without actually doing any Paediatrics is a bit of an issue.” (Doctor C)
	“…they have difficulty recognising quite straight forward childhood problems…” (Doctor D)
	All of the receiving clinicians suggested that more paediatric exposure and training for GPs may help reduce the numbers of acute medical paediatric emergency admissions.
*Views on whether a short period of observation is considered to be appropriate*	“I think if they come in and then are assessed and sent home then that is obviously an admission that didn’t need to come in and see us.” (Doctor B)
	“…here in PAU we have the opportunity for a short period of observation which often allows us to be more confident about sending a child home…” and this doctor also mentioned that “…observation is not harmful…” (Doctor E)
	“…I think it is good for the benefit of the family…” (Doctor G)
	“…we do send lots home very quickly so therefore you could argue that they didn’t need to be admitted but I think part of the just being seen, being assessed and reassured is part of the process.” (Doctor D)

## Supplementary Materials

The following are available online at http://www.mdpi.com/2227-9032/6/3/117/s1, Table S1: characteristics of referring clinicians, Table S2: characteristics of receiving clinicians, Table S3: characteristics of parents/caregivers and their child.

## Appendix A. Topic Guide for Parents

**1. Personal details**

- What is your relationship to this child?
- How old is your child?
- How old are you?
- How is the general health of your child and do you they have any medical conditions?

**2. Admission process**

- How long was your child unwell for before you decided to seek healthcare?
- Where and when did you seek healthcare for your child’s illness?
- How and when was your child admitted to hospital?
- Why did you decide it was necessary to seek healthcare for your child?
- Did you expect your child to be admitted to hospital?

**3. Personal experience**

- Thinking about the process of your child being admitted to hospital, what do you feel has been done well and is there anything you feel could have been done better?

**4. Previous experience**

- Has your child been admitted to hospital before and if so what for?
- Was there anything that you feel could have been done better in past experiences?
- Do you have any other children? If so, are they older or younger?
- If answer yes to c, do you think if this had occurred with your first or second child anything would have been different?

**5. In general**

- If you were unsure as to whether to seek healthcare for your child what would you do?
- Do you think there are general factors which may influence parents’ decision to seek healthcare for their child?
- Is there anything else you would like to tell me about the experience of your child being admitted to hospital?

## Appendix B. Topic Guide for Referring Clinicians

**1. Experience**

- What is your job title?
- How long have you been working in this role?
- How much training have you had in acute Medical Paediatrics?

**2. With respect to the case you most recently referred to hospital for acute medical assessment**

- What was the age of the child you saw?
- What was the parent’s /caregiver’s primary concern when you saw the child?
- What factors informed your decision to admit this child?
- Has past experience influenced your decision to admit this child?
- Might you have made a different decision regarding the admission of this child earlier on in your training?
- Do you think you might have made a different decision if you had additional specialist training?
- If you had seen this child on a different day of the week or time of the day, do you think your decision to admit would have been different?
- If you had seen this child from a different socioeconomic background or with a slightly different combinations of symptoms, would your decision regarding the admission of this child have been different?

**3. Confidence**

- Are there any generic triggers which make you consider admitting a child to hospital?
- Do you feel confident in spotting a sick child who requires hospital care versus one who doesn’t? If not, what might help you increase your confidence in spotting a sick child?
- If you are unsure about the diagnosis or management of a child what would you do?
- If you are uncertain as to whether to admit a child to hospital what would you do?
- Do you feel well supported by colleagues? If not, what do you think would make you feel more supported?

## Appendix C. Topic Guide for Receiving Clinicians

**1. Experience**

- What is your job title?
- How long have you been working in this role?
- How much training have you had in acute Medical Paediatrics?

**2. With regard to the most recent case you saw**

- What was the age of the child you saw?
- What was the parent’s/care giver’s primary concern when you saw the child?
- Where was the child referred from?
- Do you think that this case merited referral to hospital? If not, which other actions do you think could have been taken in this case?

**3. In general**

- Why do you think there are rising numbers of children admitted to hospital as emergencies, with acute medical conditions, and for shorter durations?
- Do you think there any particular factors that may increase the likelihood of a child being admitted to hospital?
- Why do you think that some children are admitted to hospital unnecessarily, with conditions which could be managed at home or in the community?
- How would you define an inappropriate acute medical paediatric emergency admission?
- How would you define a serious acute medical paediatric emergency admission?
- Do you think there is anything that could, or should be done to safely reduce acute medical paediatric emergency admissions?
