# Supplementary material for: Physician and Parental Decision—Making Prior to Acute Medical Paediatric Admission
Source: Healthcare (Basel). 2018 Sep 17;6(3):117. doi: 10.3390/healthcare6030117 (PMC6165442; doi:10.3390/healthcare6030117)
Supplement: Supplementary file 1 [file healthcare-06-00117-s001.docx]

**Table S1. Characteristics of referring clinicians.**

|  | **Job title** | **Length of time working in role** | **Training in acute medical paediatrics** | **Age of child** | **Parent/caregiver’s primary concern** | **Past experience** | **Different decision earlier in training** | **Different decision additional specialist training** | **Confident in spotting sick child** | **Feel well supported by colleagues** |
| --- | --- | --- | --- | --- | --- | --- | --- | --- | --- | --- |
| **Doctor A** | Registrar in A+E | 6 months | 9-12 months | 4 years old | Level of consciousness | yes | no | no | yes | yes |
| **Doctor B** | GP | 26 years | 2 months | 2 years old | Acutely unwell | yes | no | no | Would hope so | yes |
| **Doctor C** | GP | 9 years | None | 18 months | Breathing problems | yes | no | no | Yes | Yes in GP practice, less by hospital |
| **Doctor D** | G-MED doctor | 2 and a half years | 9 months | 20 months | Breathing problems | yes | no | not sure | To an extent | yes |
| **Doctor E** | G-MED doctor | 11 years | None | 24 months | Fever and shaking | yes | no | no | Hope so | yes |
| **Doctor F** | ST4 in A+E | 8 months | None | 17 months | Allergic reaction | No | No | Don’t think so | Yes | Yes |
| **Doctor G** | Registrar in A+E | 8 years | None | 2 years old | seizure | yes | no | Not sure | yes | yes |

**Table S2. Characteristics of receiving clinicians.**

|  | **Job title** | **Length of time working in role** | **Training in acute medical paediatrics** | **Age of child** | **Parent/caregiver’s primary concern** | **Route of referral** | **Merited referral** |
| --- | --- | --- | --- | --- | --- | --- | --- |
| **Doctor A** | FY1 | 2 months | Weeks | 5 months old | fever, coughing and reduced feeding | not available | not sure, not expect to stay over night |
| **Doctor B** | Paediatric registrar | 3 years | 4 and a half years | 7 years old | breathing problems | GP | no- all needed was GP to reassure parents |
| **Doctor C** | ST1 in paediatrics | 1 month | 4 months | 12 years old | constipation | GP | yes |
| **Doctor D** | Associate specialist | 4 years | 24 years | 13 years old | headache, dizzy and generally unwell | GP | yes |
| **Doctor E** | Medical paediatric registrar | Not available | 9 years | 6 months | temperature, mottled skin and worried about something serious | A+E | yes |
| **Doctor F** | Locum paediatric registrar | 5 years | 14 years | not available | difficulty breathing | not available | yes |
| **Doctor G** | ST1 in paediatrics | 7 months | Not available | 18 months old | worried that child had been unwell for a couple of months | GP and ambulance | yes |
| **Doctor H** | Locum paediatric SHO and GP registrar | 1 and a half months | 3 and a half years | 15 years old | blurry vision and headache | A+E through advice from GP | yes |
| **Doctor I** | FY1 | 7 months | 4 months | not available | bloody stools | GP | no-part of history missed |
| **Doctor J** | FY1 | 6 months | 4 months | 2 years old | not passing urine | GP | yes |

**Table S3. Characteristics of parents/caregivers and their child.**

|  | **Relationship to child** | **Age of child** | **Age of parent** | **General health of child** | **How long unwell for** | **Where sought healthcare** | **Expect to be admitted** | **Admitted in past** | **Other children** |
| --- | --- | --- | --- | --- | --- | --- | --- | --- | --- |
| **Parent A** | Mum | 3 and a half years old | 40 years old | normally well | 2 days | Phoned 999 ambulance service | yes | yes | No |
| **Parent B** | Dad | 13 months old | not available | normally alright | 1 and a half days | GP | no | yes | No |
| **Parent C** | Mum | 1 and a half years old | 21 years old | good | Precaution- straight away | NHS 24 then A+E | Did but didn’t | no | No |
| **Parent D** | Mum | 4 and a half years old | 35 years old | Tetralogy of fallot | 3 days | GP first then G-DOCS | yes | yes | Not available |
| **Parent E** | Mum | 10 years old | 35 years old | normally well | 2 days | NHS 24 then A+E | Wasn’t sure, had suspicions | no | 2 others |
| **Parent F** | Mum | 10 years old | 39 years old | Imperforate anus, floppy windpipe and oesphagitis | 1 day | A+E | no | yes | 1 older |
| **Parent G** | Mum | 5 weeks old | 39 years old | Good, preterm | Wasn’t unwell, was advised by GP | A+E | no | No- except in neonatal because preterm | 1 older |
| **Parent H** | Mum | 10 months old | 24 years old | Narrow airways in nose | 3 days | GP | yes | yes | 1 younger |
| **Parent I** | Mum | 9 years old | 32 years old | Asthma | 1 day | A+E | Not sure | yes | 1 younger |
| **Parent J** | Mum | 3 years old | 36 years old | Normally well | 12 hours | GP | Knew was a good chance | yes | 1 older |
